# Insights into Human Middle Ear Implants: Uncovered Bistability

**DOI:** 10.3390/ma17235730

**Published:** 2024-11-23

**Authors:** Robert Zablotni, Grzegorz Zając, Rafal Rusinek

**Affiliations:** 1 Department of Applied Mechanics, Mechanical Engineering Faculty, Lublin University of Technology, Nadbystrzycka 36, 20-618 Lublin, Poland; r.zablotni@pollub.pl (R.Z.); r.rusinek@pollub.pl (R.R.); 2 Department of Power Engineering and Transportation, Faculty of Production Engineering, University of Life Sciences in Lublin, Głeboka 28, 20-612 Lublin, Poland

**Keywords:** middle ear dynamics, implantable middle ear hearing device, bistability

## Abstract

This study delves into the intricate mechanics of human middle ear implants by examining a lumped parameter model with five degrees of freedom to estimate sound transfer. The ASTM standard, recognized globally as a benchmark, served as a reference for analysis, ensuring test accuracy and providing a comprehensive evaluation framework. To assess the implant’s usability, numerical simulations were conducted and compared against both the ASTM standard and the experimental results obtained from temporal bone studies. This investigation uncovered the bistability of periodic responses induced by the implant, prompting an analysis of the bistability in periodic solutions and the creation of basins of attraction for various initial conditions. The discovery of new solutions underscores this study’s significance in the operation and reliability of implants. Consequently, this research not only enhances the theoretical comprehension of the system, but also holds promise for practical applications in the design and optimization of middle ear implants that transfer energy to the stapes and the cochlea.

## 1. Introduction

The human auditory faculty exists among the main senses, sometimes requiring assistance from external (Air Conduction Hearing Aids) or internal (Implantable Middle Ear Hearing Devices—IMEHDs, Bone-Anchored Hearing Aids—BAHAs, or Cochlear implants) instruments to enhance hearing in the case of partial or complete hearing loss, both in the cases of conductive and sensorineural hearing loss. The upkeep and dependability of such tools must adhere to stringent standards to guarantee sustained efficacy. The human auditory system allows us to perceive and distinguish the vast range of sounds that surround us. The human auditory system stands as a testament to complex design, involving soft tissues, bones, ligaments, and tendons, as well as an organ that transmits a nerve impulse to the brain, thus enabling us to discern the diverse array of sounds enveloping us in the world. Its reliability is unmatched. Central to this auditory journey is the intricate process of hearing, which is akin to a symphony orchestrated by the delicate structures of the ear. The passage of sound from the external environment to our perception entails multiple stages, with the middle ear assuming a pivotal role in molding and conveying of auditory signals.

The ability of humans to hear involves an extraordinary combination of both physiological-like sound wave capture, vibration transmission, and fluid movement in the inner ear and neurological activities, where hair cells convert these vibrations into electrical signals sent to the brain. The major aspect of sound transmission outside the human ear is through the involvement of air. In the air, the sound wave spreads and reaches the eardrum, which begins to vibrate. Essentially, a mechanical wave is captured by the auricle and directed by the external auditory canal to arrive at the tympanic membrane (TM), which is referred to as the eardrum. When those waves hit the TM, they cause it to vibrate, triggering a chain of occurrences that lay the groundwork for our perception of sound.

The human middle ear (ME), which begins at the TM and ends at the inner ear, functions as a sophisticated transmission system. The ME include the three tiny bones known as the ossicle chain—malleus, incus, and stapes (in that order). They cooperate in order to convert the comparatively low-pressure and large-amplitude motions of the eardrum into high-pressure and smaller-amplitude vibrations, optimizing the transfer of sound energy to the inner ear.

Comprehension of the anatomical structure of the middle ear is important for unraveling the complexities of auditory processing. The malleus is connected to the TM and by the IMJ with the incus, which is connected by the ISJ to the stapes. The stapes is connected to the oval window of the cochlea, which is filled with fluid and is located in the inner ear. The inner ear is responsible for converting mechanical vibrations into electrical signals that can be interpreted by the brain.

The ME also includes the Eustachian tube, which plays a key role in balancing the pressure between the middle ear and the outside world. This function is essential for preserving ideal conditions for hearing and avoiding the discomfort that can arise from differences in pressure.

The quest to understand the intricate workings of the ME and its role in auditory processing has driven scientists and researchers to develop models that simulate motion. The history of modeling the human middle ear can be traced back to the early anatomical studies conducted by pioneers like Leonardo da Vinci, who laid the foundation for comprehending the complexities of the ear’s internal structures [1].

The inception of research into human middle ear modeling can be traced back to 1961 with Mőller’s pioneering study [2], which introduced a novel scheme for the middle ear mechanism. Following this, Zwislocki [3] explored a similar model, both drawing inspiration from Bárány’s widely accepted theory, which posits that the ossicles rotate about the axis passing through the head of the malleus, and the short process of the incus. Zwislocki, in particular, assumed a rigid coupling between the malleus and the incus. In subsequent decades, there has been a shift toward developing mechanical models that utilize lumped masses. In these models, the ossicles—comprising the malleus, incus, and stapes—are represented as rigid bodies that are interconnected by springs and dampers. Notably, for low-excitation frequencies governing speech recognition, the stapes motion is likened to a piston [4], leading to the prevalent consideration of planar motion in literature-reported models. Models of the ME vary in complexity, ranging from three [5] to four [6] and even six [7] DOF. Typically, these models concentrate on the kinematic relationships within the intact middle ear. In contrast, Feng and Gan [7] delved into the dynamic aspect of the middle ear, conducting numerical analyses that demonstrated a noteworthy alignment with experimental findings. It is worth noting that the aforementioned models are predominantly linear, with joints and ligaments represented as Kelvin–Voigt (K-V) elements. This linear characterization, however, simplifies the true nature of the ear as ligaments, joints, and tendons exhibit nonlinear behavior, as documented in the literature [8]. Models of the human middle ear serve as invaluable tools for biomechanics and otolaryngologists alike. They provide insights into the intricate mechanics of the middle ear, allowing for a deeper understanding of how various factors influence auditory function. These models enable researchers to simulate and analyze the impact of different conditions, such as diseases or structural abnormalities, on hearing capabilities. Additionally, middle ear models play a crucial role in the design and refinement of hearing aids and other auditory devices. By replicating the complex interactions within the middle ear, engineers can optimize the performance of hearing aids, ensuring that they effectively amplify sound while minimizing distortion.

One of the most significant advancements in auditory technology is the development of implantable devices that directly interface with the middle ear. Floating mass transducers (FMTs) are a notable example of such devices. FMTs are miniature implants that leverage the principles of mass-spring systems to directly stimulate the ossicles and enhance sound transmission. Implantable middle ear devices, including FMTs, offer a promising solution for individuals with hearing impairments. By bypassing certain deficiencies in the natural auditory pathway, these devices can provide enhanced hearing experiences and improved quality of life for individuals with various degrees of hearing loss. Implantable devices also have certain disadvantages, particularly in relation to surgical risks and complications. Middle ear implants (MEIs) necessitate a surgical procedure for implantation, which inherently carries risks such as infection, tissue inflammation, and potential adverse reactions to anesthesia. In some cases, patients may experience damage to the ossicular chain, facial nerve injury, or other complications associated with the specific location of the implant. Additionally, post-surgical issues like device extrusion or migration can arise, sometimes requiring further revision surgeries to address these complications. Moreover, electromagnetic implants are susceptible to interference from external electromagnetic fields, which may affect their performance and lead to unwanted noise or signal distortion. Therefore, piezoelectric device are increasingly popular these days.

The response of the middle ear to an IMEHD has been standardized and is described in ASTM standard no F2504-05—Standard Practice for Describing System Output of Implantable Middle Ear Hearing Devices. However, from a maintenance and reliability point of view, the IMEHD should also be tested at different dynamical conditions.

In this work, the model of the IOC [9] was extended to five degrees of freedom (DOF) by including two masses that composed a middle ear implant attached to the incus. Unlike the results presented in the ASTM standard [10], this work determined the bistability of the periodic response stimulated by an implant. Therefore, an analysis of the bistability of periodic solutions was performed, and basins of attraction for different initial conditions (ICs) were created. The expected response of the ear should be clearly identified so that it sounds the same. In the case of bistability, the sound can be heard in different ways. The user expects a clearly identified response during the hearing process, which can be disrupted in the case of bistability. The aim of this work was to demonstrate that bistability, which appears in various nonlinear systems, is an undesirable phenomenon in the context of human hearing. Therefore, this study explains when it may occur in an implanted middle ear system. This research was conducted from the point of view of the operation and the reliability of the implant. The research of middle ear implants according to world standards ensures correct and long-time operations.

The structure of this paper will be in the following manner: Section 2 describes the experimental procedure from [9,11], the data from ASTM standard [10], and the explanation of the mathematical model of the implanted human middle ear developed from [9]. Subsequently, in Section 3, this paper focus on the outcomes of the numerical analysis and the experimental result comparisons of the stapes vibrations, thereby exploring the frequency response function, the impacts of the excitation applied on the implant, the bistability in the response function, and the basin of attraction for various ICs. Finally, Section 4 includes a discussion of the results and conclusions.

## 2. Materials and Methods

The methodology of this experimental research on a temporal bone was conducted in accordance with the ASTM F2504-05 standard [10]. Therefore, this section includes a brief summary of the ASTM standard, and the results are also presented therein. Additionally, a procedure of a temporal bone preparation and testing is described. The ASTM F2504-05 standard is a widely recognized benchmark in the field of the middle ear biomechanics; therefore, it is used as a reference point for comparison. This standard provides a rigorous set of criteria for assessing the quality of measurements and methods. The ASTM standard was created based on Rosowski’s work [12], which is one of the most recognizable works on measurements inside the middle ear. In addition, there are recommendations contained in the world literature on adhering to this standard to assess the correctness of the measurement procedures used.

### 2.1. Standard Procedure

In accordance with the ASTM F2504-05 standard, Implantable Middle Ear Hearing Devices (IMEHDs) offer alternatives to air conduction hearing aids. They share similarities with air conduction hearing aids in processing incoming sound through the application of frequency shaping and compression to generate an analog, vibratory audio frequency output. Notably, IMEHDs diverge from hearing aids by employing a distinctive mechanism; instead of creating an airborne acoustical output signal with an electroacoustic output transducer in the external ear canal, they utilize mechanical stimulation, resulting in the vibration of the cochlear fluid. As a consequence of this unique approach, the output signal produced by IMEHDs is not easily accessible post-implantation, unlike hearing aid output, which can be readily measured using real-ear probe microphone techniques. Therefore, research on IMEHD application is especially required.

The ASTM standard [10] establishes consistency in data collection practices, facilitating the evaluation and direct comparison of IMEHD performances. As clinical data become accessible, performance specifications can be enhanced by incorporating corresponding transfer functions or measurement results obtained from patients. The temporal bone serves as a widely accepted model that is closely aligned with the biomechanics of the living middle ear, making it directly applicable to hearing level considerations. A Laser Doppler vibrometer offers precise velocity measurements within the ranges essential for human hearing [4,10,12,13,14,15,16]. The experimental results presented in the ASTM standard [10] were derived from 10 publications. Among these, 9 of the publications focused on measuring the velocity of the stapes footplate, which is connected to the oval window using LDV, while 1 publication specifically measured the displacement of the round window. The results from the ASTM standard are shown in Table 1, and they were used as the reference value in many papers [4,15,16,17]. 

### 2.2. Temporal Bone Preparing

**Figure 1:**
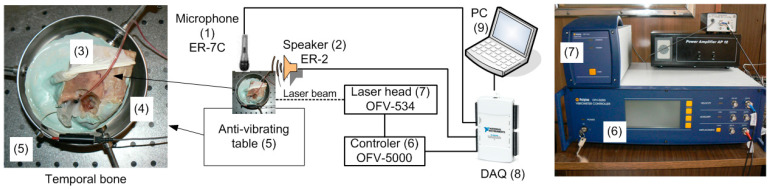
Scheme of the experimental setup used in the temporal bone experiment.

To ascertain the transfer function of the middle ear (ME) and validate its model, a series of experiments were conducted using six human temporal bone specimens. These specimens underwent standard otolaryngology procedures that involved the removal of soft tissue followed by antromastoidectomy with posterior tympanotomy. The mastoid facial nerve was removed to enable visualizations of the stapes arch and footplate. An artificial external ear canal, measuring 25 mm in length and 9 mm in diameter, was then securely attached to the bone using epoxy resin. This canal featured two ports, as depicted in Figure 1 and elaborated in [9,11]: one for an ear microphone (ER-7C Etymotic Research) and another for a sound source (the speaker used in ER2 Etymotic Research).

The artificial canal was sealed with a glass plate, forming a sound-sealed chamber. Reflective tape pieces, each weighing less than 0.05 mg, were strategically positioned on the footplate of the stapes. Subsequently, the temporal bone specimen was embedded in dental cement to fix in the temporal bone holder by Storz. This cement did not interfere in the experimental results because it was used outside the measurement area, and it was only used to fix the temporal bone. Measurements were conducted on an anti-vibration table within a sound booth. Middle ear stimulation involved applying a discrete chirp signal with a frequency range from 0.25 to 8 kHz to capture the ear characteristics within the hearing range. Similar setups for the measurements, as well as experimental results, are shown in [17,18].

### 2.3. Temporal Bone Testing

The velocity of the stapes footplate was measured using a LDV system manufactured by Polytec GmbH, Waldbronn, Germany. This system consisted of a Polytec controller, OFV-5000, and a laser head, OFV-534. The He-Ne laser beam was directed at retro-reflective markers situated on the stapes footplate through the artificial ear canal. The measured stapes velocity was transmitted from the controller to the National Instruments DAQ card NI6210 (National Instruments, Austin, TX, USA) and then routed through the PC to the DasyLab software (DasyLab12) for data capture (refer to Figure 1). The Polytec VD-06 decoder used in these tests possesses a very high accuracy of velocity measurements at 0.01 μm/s. Experimental data using LDV were obtained by reflecting a laser beam onto a reflective tape attached to the stapes footplate.

The frequency response function (FRF) of the middle ear stands as a cornerstone tool for researchers across engineering and medical disciplines. This physical representation encapsulates the relationship between a system’s output and input. Typically, the applied pressure on the ear constitutes the input, while the resultant stapes vibration velocity induced by this pressure serves as the output. In this context, we compared the FRF derived from the ASTM standard and experiments outlined in [9,19] with the numerical results obtained for the 5-DOF human middle ear model implant presented in this study. The FRF is defined as an acousto-vibrational transfer function, and it is expressed as follows:

(1) $$H_{Tv}=\frac{v}{p_{T}}$$

where:

$p_{T}$—TM sound pressure;
v—stapes footplate velocity.

The pressure on the tympanic membrane is an important factor in the process of sound conduction. The velocity of the stapes vibration is proportional to the pressure, as shown by the works of [4,15,16].

## 3. Model of the Human Middle Ear with Implant

**Figure 2:**
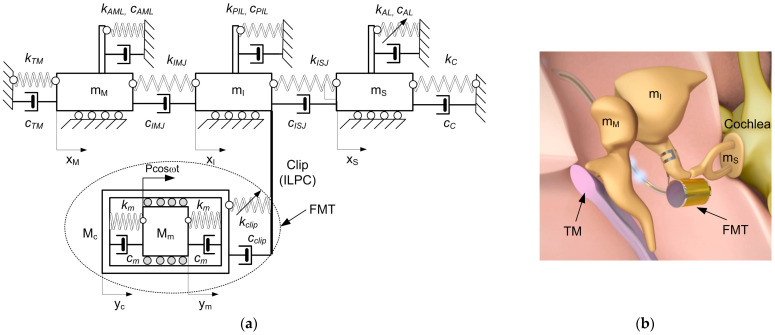
(**a**) A 5-DOF LPM with a floating mass transducer and mechanical excitation; (**b**) view of the implanted human middle ear [www.medel.pro].

In the human middle ear, the connection between the malleus and the TM is established through the handle of the malleus, while the attachment to the temporal bone involves the lateral mallear ligament (LML), the tensor tympani tendon (TTT), and the anterior mallear ligament (AML). Situated in the epitympanic recess, the head of the malleus articulates with the incus via the icudomallear joint (IMJ). The incus, comprising a body and two processes, articulates with the malleus through the body, attaches its short process to the posterior wall of the middle ear, and connects its long process to the stapes through the incudostapedial joint (ISJ). Ligaments such as the superior mallear ligament (SML) and the posterior incudal ligament (PIL) secure the incus to the bone. The stapes, the smallest bone in the human body, links the incus to the oval window of the inner ear. The footplate of the stapes is connected to the oval window through the annular ligament (AL) and to the temporal bone through the stapedial tendon (ST). Two protective muscles in the middle ear are the tensor tympani and the stapedius muscle. The stapedius muscle contracts in response to loud noises, inhibiting ossicle vibrations and reducing sound transmission to the inner ear—an effect known as the acoustic reflex. While modeling the sound transmission process in this study, the acoustic reflex is disregarded. A similar model to the one in Figure 2 can be found in [11,20]. In this case, however, it was different because the implant was excited by force and not by electricity as in the case in [11].

The proposed middle ear (ME) model involves masses connected by springs (*k*) and dampers (*c*). The annular ligament (AL) exhibits nonlinear (cubic) stiffness characteristics (*k_AL3_*), as reported in [8]. Three masses represent the ossicles, allowing horizontal movement on a base [9]: the malleus (*m_M_*), the incus (*m_I_*), and the stapes (*m_S_*). The FMT comprises a floating mass (magnet, *M_m_*) and a metal case (*M_c_*). Suspended with dampers (*c_m_*) and silicon springs (*k_m_*), the floating mass is excited by an electromagnetic field, resulting in its movement by an external force with amplitude P and frequency (*ω*), as depicted in Figure 2a. There is a simplification that changes the electrical excitation that is usually in FMT to the mechanical excitation used in this model. Attached to the incus long process, the FMT is fixed with a coupler (clip), featuring damping and stiffness coefficients that are denoted as *c_CLIP_*, *k_CLIP_*, *k_CLIP2_*, and *k_CLIP3_*. Most of the damping and stiffness parameters used in this model are crucial for sound conduction. These parameters replace the key ligaments and tendons. The coupler’s stiffness is assumed to be nonlinear, and it is adaptable to individual patient needs, which is important from a maintenance and reliability point of view. Consequently, the governing differential equations of the implanted human middle ear system in dimensional form are defined as follows.

(2) $${\ddot{x}}_{M}m_{M}+{\tilde{k}}_{11}x_{M}+{\tilde{k}}_{12}x_{I}+{\tilde{c}}_{11}{\dot{x}}_{M}+{\tilde{c}}_{12}{\dot{x}}_{I}=0,$$

(3) $$\begin{array}{c} {\ddot{x}}_{I}m_{I}+{\tilde{k}}_{21}x_{M}+{\tilde{k}}_{22}x_{I}+{\tilde{k}}_{23}x_{S}+{\tilde{k}}_{24}x_{c}+{\tilde{\gamma }}_{24}{\left(x_{I}-x_{c}\right)}^{3}+{\tilde{\beta }}_{24}{\left(x_{I}-x_{c}\right)}^{2}+\\ {\tilde{c}}_{21}{\dot{x}}_{M}+{\tilde{c}}_{22}{\dot{x}}_{I}+{\tilde{c}}_{23}{\dot{x}}_{S}+{\tilde{c}}_{24}{\dot{x}}_{c}=0, \end{array}$$

(4) $${\ddot{x}}_{S}m_{S}+{\tilde{k}}_{32}x_{I}+{\tilde{k}}_{33}x_{S}+{\tilde{c}}_{32}{\dot{x}}_{I}+{\tilde{c}}_{33}{\dot{x}}_{S}+{\tilde{\gamma }}_{3}{x_{S}}^{3}=0,$$

(5) $$\begin{array}{c} {\ddot{x}}_{c}M_{c}+{\tilde{k}}_{42}x_{I}+{\tilde{k}}_{44}x_{c}+{\tilde{k}}_{45}x_{m}+{\tilde{c}}_{42}{\dot{x}}_{I}+{\tilde{c}}_{44}{\dot{x}}_{c}+{\tilde{c}}_{45}{\dot{x}}_{m}-{\tilde{\gamma }}_{24}{\left(x_{I}-x_{c}\right)}^{3}-\\ {\tilde{\beta }}_{24}{\left(x_{I}-x_{c}\right)}^{2}=0, \end{array}$$

(6) $${\ddot{x}}_{m}M_{m}+{\tilde{k}}_{54}x_{c}+{\tilde{k}}_{55}x_{m}+{\tilde{c}}_{54}{\dot{x}}_{c}+{\tilde{c}}_{55}{\dot{x}}_{m}=Pcos\left(\omega t\right).$$

The system coefficients are defined as follows:

(7) $$\begin{array}{c} {\tilde{k}}_{11}=k_{TM}+k_{AML}+k_{IMJ},{\tilde{k}}_{12}=-k_{IMJ},{\tilde{c}}_{11}=c_{TM}+c_{AML}+c_{IMJ},\\ {\tilde{c}}_{12}=-c_{IMJ},{\tilde{k}}_{21}={\tilde{k}}_{12},{\tilde{k}}_{22}=k_{PIL}+k_{ISJ}+k_{IMJ}+k_{clip},\\ {\tilde{k}}_{23}=-k_{ISJ},{\tilde{k}}_{24}=-{\tilde{k}}_{clip},{\tilde{\gamma }}_{24}=k_{clip3},{\tilde{c}}_{21}=-c_{IMJ},\\ {\tilde{c}}_{22}=c_{PIL}+c_{ISJ}+c_{IMJ}+c_{clip},{\tilde{c}}_{23}=-c_{ISJ},{\tilde{c}}_{24}=-c_{clip},\\ {\tilde{k}}_{32}={\tilde{k}}_{23},{\tilde{k}}_{33}=k_{AL}+k_{ISJ}+k_{C},{\tilde{c}}_{32}={\tilde{c}}_{23},{\tilde{c}}_{33}=c_{AL}+c_{ISJ}+c_{C},{\tilde{\gamma }}_{3}=k_{AL3},{\tilde{k}}_{42}={\tilde{k}}_{24},\\ {\tilde{k}}_{44}=k_{clip}+k_{m},{\tilde{k}}_{45}=-k_{m},{\tilde{c}}_{42}={\tilde{c}}_{24},{\tilde{c}}_{44}=c_{clip}+c_{m},{\tilde{c}}_{45}=-c_{m},{\tilde{\gamma }}_{24}=k_{clip3},\\ {\tilde{\beta }}_{24}=k_{clip2},{\tilde{k}}_{54}={\tilde{k}}_{45},\\ {\tilde{k}}_{55}=k_{m}=-{\tilde{k}}_{54},{\tilde{c}}_{54}={\tilde{c}}_{45},{\tilde{c}}_{55}=c_{m}=-{\tilde{c}}_{54}. \end{array}$$

Drawing from the mathematical models described earlier, the numerical models were formulated using MATLAB R2020b Simulink. Subsequently, numerical computations were performed using the Runge–Kutta fourth-order method (ode45), with a variable step size and a relative tolerance of 10^−10^. The system is described by classical nonlinear equations; therefore, the Runge–Kutta method was chosen, which is well developed in MATLAB and provides a satisfactory accuracy of results in a relatively short time. The relative tolerance and variable integration step used are related to the stapes displacements, which are nanometers in size. The numerical experiments entail employing the parameters outlined in Table 2.

The stiffness (*k*), damping (*c*), and mass (*m*) parameters were obtained from [14], while the remaining parameters introduced in the models were determined through estimation based on experimental findings. This approach ensures that the numerical outcomes, particularly the FRF, align with the corresponding experimental results.

## 4. Result Comparisons

The analysis placed particular emphasis on stapes vibration due to its crucial role in facilitating the sound transmission to the inner ear. Therefore, configuring and operating an implant is of utmost importance from an end user’s perspective. The numerical simulations presented in this study, along with the results of the experiments conducted on temporal bones, suggest that the outcomes reported in the ASTM standard, which are recommended for IMEHD testing, are not always accurate. Initially, the ASTM standard was compared to the experimental findings on the intact ossicle chain (IOC), and this was followed by comparisons with the numerical simulations conducted for both the 3 (IOC) and 5 degrees of freedom (DOF) model of the implanted ME.

### 4.1. ASTM Standard and Experiment

**Figure 3:**
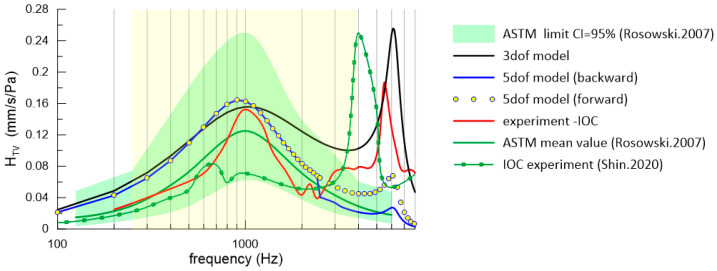
Comparison of the experimental and numerical results expressed by the transfer function (HTV) with ASTM standard and literature data [12,22].

The green area in Figure 3 depicts the range of results within the ASTM standard, with the green curve representing the mean values of 10 measurements.

According to the ASTM standard [10], a single resonance occurs around 1 kHz within the range of values spanning from 0.250 to 0.062 mm/s/Pa. Beyond 1 kHz, the curves gradually decrease up to 6 kHz, as shown in Figure 3. In the ASTM standard results, normalization was performed to a pressure of 1 Pa, which is an equivalent of 94 dB SPL.

Experimentally measured stapes vibration, as reported in [9], is illustrated in Figure 3. The experimental findings highlight that the primary resonance of the intact middle ear occurs around 1 kHz, and it is followed by a second resonance at approximately 5.5 kHz, which is not shown in the ASTM standard. However, the results of our experiment fall within the tolerance range recommended by the ASTM standard (green area), and they also fall within in a frequency range that is also recommended by the ASTM standard (yellow area 250–4000 Hz). In the case of the 3-DOF IOC model results, there was also a second resonance peak, but bistability was not detected since the bistability was only caused by the implant (FMT). Moreover, similar results can be found in [17,18,21]. In the works of [22,23], there is also a second resonance peak at a frequency of 4 kHz. The existence of an additional peak (5.5 kHz) is numerically verified in the next subsection. The difference between the IOC results from [9,22], and this study may be due to the difference in the weight of the reflective tapes that changes the mass of the ossicles. Moreover, some of the differences between the anatomy of the temporal bones have been observed in the literature.

In both the numerical and experimental results, there were two resonance peaks. The first key resonance peak occurred at a frequency of 1 kHz. The second resonance peak, depending on the research and the model, occurs in the frequency range from 4 to 6 kHz. In the case of the model with the 5-DOF implant, the second resonance peak was significantly lower than in the other cases with respect to the experimental results of the IOC and the 3-DOF IOC model.

### 4.2. Numerical Simulation Results

**Figure 4:**
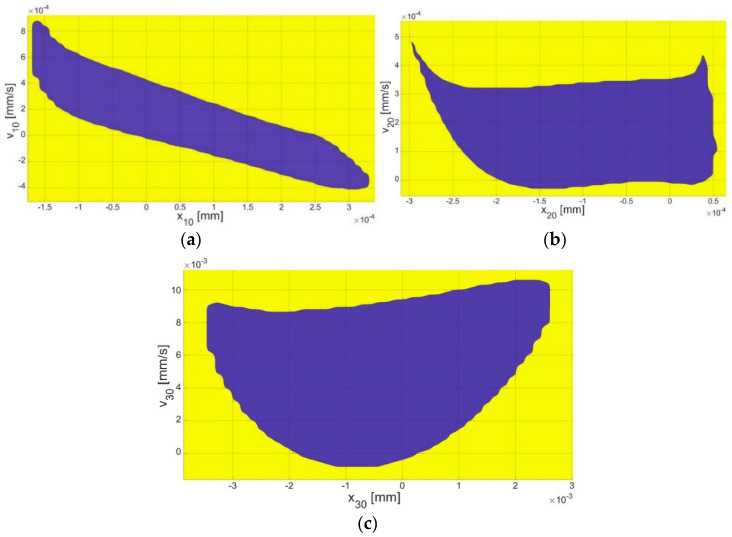
The basin of attraction for the stapes motion of the initial conditions of the (**a**) malleus (x_10_, v_10_), (**b**) incus (x_20_, v_20_), and (**c**) stapes (x_30_, v_30_).

**Figure 5:**
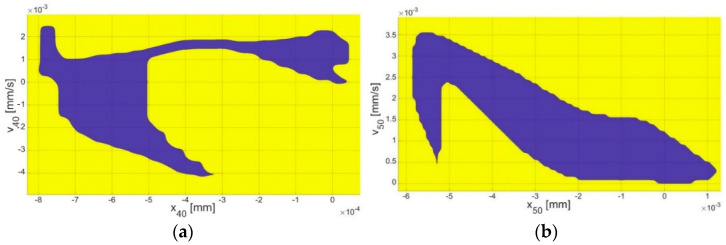
The basin of attraction for the stapes motion of the initial conditions of the (**a**) clip of the implant (x_40_, v_40_), and the (**b**) floating mass (x_50_, v_50_).

The numerical simulation results for the IOC (3-DOF model), which is depicted as the black curve in Figure 3, were juxtaposed with the outcomes of the experiment on the IOC to verify the existence of an additional (second) resonance peak (5.5 kHz). Consequently, the numerical confirmation of the second resonance was substantiated. Furthermore, the presence of the second resonance has been documented in [22] (green curve with markers). This sheds new light on the ASTM standard, which fails to account for all the potential cases of stapes resonances. Moving forward, IMEHDs should also be tested in terms of identifying the second resonance. Interestingly, the nonlinear model of IME (5-DOF) presented here demonstrates a possibility of there being periodic solution bistability near the second resonance (as illustrated by the blue curve and yellow dots in Figure 3), which is a phenomenon that is not addressed in the ASTM standard but has been mentioned in some papers dedicated to IMEHDs [24,25]. In this scenario, the main resonance peak was also observed at 1 kHz, which is within the ASTM limit. Notably, there was a bifurcation of the curves above 2.5 kHz, where the blue curve and yellow dots split into two branches (lower and upper). Thus, two periodic solutions were found to be possible depending on the initial conditions (ICs). Consequently, an analysis of the ICs was essential to identify the basins of attraction (Figure 4 and Figure 5).

Under small initial conditions (the blue area in Figure 4 and Figure 5), the resonance curve fell within the range described by the ASTM standard (blue curve in Figure 3). However, when larger initial conditions (the yellow area in Figure 4 and Figure 5) were considered, the values of the H_TV_ increased for frequencies above 2.5 kHz, exceeding the ASTM standard values (the yellow points in Figure 3). Therefore, investigating the impact of the initial conditions, including the displacement (*x*) and velocity (*v*) on the system response, was crucial.

Numerical calculations of the basin of attraction (Figure 4 and Figure 5) were conducted for frequency *ω* = 2.5 kHz. Each mass was examined for their initial conditions. The magnitude of the initial conditions for the first (*x_10_*, *v_10_*) and second (*x_20_*, *v_20_*) mass (Figure 4a,b) corresponding to the malleus and incus was at the order of 10^−4^ mm, while, for the stapes (Figure 4c) (the third mass, *x_30_*, *v_30_*), it was at the order of 10^−3^ mm. In the case of the FMT mass (Figure 5a,b), the magnitude of the initial conditions (*x_40_*, *v_40_* and *x_50_*, *v_50_*) was also around 10^−3^ mm. For the implant, the velocity-related initial conditions were of the same order, 10^−3^ mm, while the displacement-related initial conditions were at the order of 10^−4^ mm.

### 4.3. Bistability Analysis

**Figure 6:**
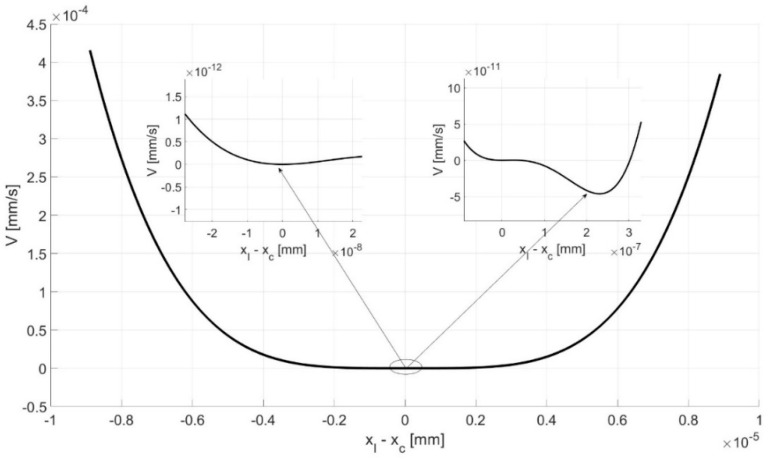
Influence of the clip stiffness on the bistable responses of the 5-DOF middle ear model.

The parameters *k_clip_*, *k_clip_*_2_, and *k_clip_*_3_ represent the stiffness of the FMT clip. Among these, *k_clip_*_2_ and *k_clip_*_3_ were particularly indicative of the nonlinear characteristic and were suspected of causing bifurcation and bistability. Therefore, Figure 6 offers a visual depiction of the potential (*V*) of the clip force (*F_clip_*), which is calculated as follows:

(8) $$F_{clip}=k_{clip3}{\left(x_{I}-x_{c}\right)}^{3}+k_{clip2}{\left(x_{I}-x_{c}\right)}^{2}+k_{clip}(x_{I}-x_{c}),$$

(9) $$F_{clip}=\frac{dV}{d(x_{I}-x_{c})},$$

(10) $$dV=F_{clip}d{(x}_{I}-x_{c}),$$

(11) $$V=\frac{1}{4}k_{clip3}{\left(x_{I}-x_{c}\right)}^{4}+\frac{1}{3}k_{clip2}{\left(x_{I}-x_{c}\right)}^{3}+\frac{1}{2}k_{clip}{\left(x_{I}-x_{c}\right)}^{2}$$

The potential curve depicted in Figure 6 reveals two distinct potential well recognized as a source of bistability. Bistability, in this context, refers to the system’s ability to rest in two separate stable states (potential wells), which correspond to two different solutions (with two different amplitudes). This is a critical characteristic of many physical systems and can have significant implications for the implanted human middle ear behavior and response to external stimuli.

The fact that the nonlinear stiffness of the clip, as represented by *k_clip_*_2_ and *k_clip_*_3_, has a significant impact on the bistability of the middle ear model with five degrees of freedom is a key finding. This suggests that the clip’s stiffness and, in particular, its nonlinear characteristics play a crucial role in the system’s dynamic behavior. This insight could have important implications for the design and optimization, as well as the maintenance, of the middle ear implants in the future. It underscores the importance of considering nonlinear effects in the analysis and design of human middle ear implants.

## 5. Discussion and Conclusions

The ASTM standard delineates the acceptable range of FRF accuracy for IOC vibration that are stimulated by sound and are measured by the LDV system. Typically, a resonance peak value is observed at 1 kHz, which is also the main middle ear resonance. Notably, both Rusinek et al. [9] and Shin et al. [22] identified a resonance peak not only at 1 kHz, but also within the 4 to 6 kHz range, and this has not been reported in the newest literature [26]. This is caused by the bistability of a dynamical system. Bistability means that the system has two stable equilibrium states depending on the initial conditions. A bistable structure can rest in either of two states; consequently, it can vibrate near equilibriums. Numerical simulations have demonstrated that, in near-zero initial conditions, the FRF adheres to the ASTM standard. However, introducing initial conditions beyond zero causes the second resonance peak to exceed the acceptable limits reported in the paper by Rosowski [12] and in the ASTM standard [10]. This suggests that the non-zero initial conditions during experimental measurements, as in [9,22], may elevate the second resonance peak and increase the energy transfer from the implant to the cochlea.

The bistability in the middle ear has rarely been discussed in the literature. Only the paper of [25] has reported this phenomenon, but it was found to be driven by different mechanisms when compared to this study. Bistability has also been observed in paper [25], but it was probably induced by a piezoelectric element of the FMT, which has been most popular recently [27]. The authors included a graph showing the relationship between bistability, voltage, and frequency. The basins of attraction obtained here indicate the IC ranges for achieving specific solutions. For the zero initial conditions for all the bone elements and the implant, the system response takes the lower response. Taking into account Figure 4 and Figure 5, the values of the system response took higher solutions. Figure 6 shows the potential wells where double responses of the system can occur. Bistability in the context of hearing is an undesirable phenomenon because it causes two responses in the stapes depending on the initial conditions. This can result in an inconvenience for the end user, who, in different cases, may hear the same sound in two different ways. In the IMEHD design process, one should, therefore, strive to eliminate the bistability phenomenon.

On the other hand, the ASTM standard sets a test correctness range from 0.25 kHz to 4 kHz, and it does not preclude occurrence beyond this range, as seen with the second resonance peak. The identification of bistability under specific initial conditions has significant implications for middle ear implant functionality and auditory processes. The nonlinear component of the implant clip’s stiffness is crucial in determining system response and stability within a bistable context. It is pertinent to recognize that sound and human speech extends from 0.2 kHz to 8–10 kHz, surpassing the ASTM-defined range. Thus, the practical implications of these findings should encompass this broader auditory spectrum. Consequently, this study not only enriches theoretical comprehension, but also offers insights for real-world applications in middle ear implant design and optimization.

Currently, hearing loss has become a serious problem in society. Genetic predisposition can have a significant influence on hearing loss (HL) through various mechanisms that are primarily centered on genetic mutations [28]. This knowledge supports personalized treatment options, such as cochlear and middle ear implants. Taking the above into account, as well as the results in this work, one should also strive to improve implant design in such a way that they do not show the phenomenon of bistability.

This study, which used the LPM model to analyze middle ear implant performance, offers valuable insights, but it also comes with certain limitations that affect its accuracy and applicability. First, the LPM model, which simplifies complex structures into discrete elements, may not fully capture the detailed anatomical and mechanical complexities of the human middle ear. This simplification can lead to a less precise modeling of vibrational mechanics, particularly in how sound energy is transferred from the implant through the ossicles to the cochlea. Additionally, the LPM model may lack the ability to simulate individual patient variations accurately, such as differences in middle ear anatomy, tissue stiffness, or pathological conditions that could impact implant outcomes. Future research directions can cover modeling with more nonlinearities added, personalized parameters, hybrid modeling with FE methods, and integration with cochlear mechanics. These types of research hold promise for enhancing the predictive power of LPM models and in advancing MEI development. By addressing these limitations, researchers can work toward more reliable models that support improved MEI designs and, ultimately, better auditory outcomes for patients.

## Figures and Tables

**Table 1:** The results shown in the ASTM standard [10].

Frequency [Hz]	Mean Value [mm/s/Pa]	Upper Limit [mm/s/Pa]	Lower Limit [mm/s/Pa]
125	0.015	0.048	0.004
250	0.030	0.074	0.012
500	0.073	0.180	0.029
1000	0.125	0.250	0.062
2000	0.071	0.138	0.037
3000	0.043	0.094	0.020
4000	0.029	0.060	0.014
6000	0.018	0.047	0.007

**Table 2:** The parameters for 5-DOF LPM.

Stiffness Coefficients	Abbreviation	Value [N/m]
Tympanic membrane stiffness	*k_tm_*	300
Anterior mallear ligament stiffness	*k_aml_*	800
Incudomallear joint stiffness	*k_imj_*	1,000,000
Posterior incudal ligament stiffness	*k_pil_*	400
Incudostapedial joint stiffness	*k_isj_*	1350
Cochlea stiffness	*k_c_*	200
Annular ligament stiffness	*k_AL_*	623
Nonlinear stiffness of the annular ligament	*k_AL3_*	1.3 × 10^11^
Stiffness of the mass inside FMT	*k_m_*	850
Stiffness of the FMT clip	*k_clip_*	800
Second-order nonlinear stiffness of the FMT clip	*k_clip2_*	−2.2 × 10^10^
Third-order nonlinear stiffness of the FMT clip	*k_clip3_*	6.4 × 10^16^
Damping coefficients	Abbreviation	Value [Ns/m]
Tympanic membrane dumping	*c_tm_*	0.06
Anterior mallear ligament dumping	*c_aml_*	0.275
Anterior mallear ligament dumping	*c_imj_*	0.359
Incudomallear joint dumping	*c_pil_*	0.055
Posterior incudal ligament dumping	*c_isj_*	0.0079
Incudostapedial joint dumping	*c_c_*	0.0017
Annular ligament dumping	*c_al_*	0.0020
Mass inside the FMT damping	*c_m_*	0.32
Clip of the FMT damping	*c_clip_*	0.15
Masses	Abbreviation	Value [kg]
Malleus mass	*m_M_*	2.5 × 10^−5^
Incus mass	*m_I_*	2.8 × 10^−5^
Stapes mass	*m_S_*	1.78 × 10^−6^
Implant mass	*M_m_*	1.5 × 10^−5^
Implant clip mass	*M_c_*	1.5 × 10^−5^
Mechanical excitation	Abbreviation	Value [N]
Mechanical force applied on implant	*P*	1.2029 × 10^−4^

## Data Availability

The original contributions presented in the study are included in the article, further inquiries can be directed to the corresponding author.

## Abbreviations

The following abbreviations are used in this manuscript:

LDV	Laser Doppler Vibrometer
FRF	Frequency Response Function
FMT	Floating Mass Transducer (e.g., VSB)
IME	Implanted Middle Ear
IOC	Intact Ossicular Chain
LML	Lateral Mallear Ligament
LPM	Lumped Parameter Model
PIL	Posterior Incudal Ligament
SML	Superior Mallear Ligament
AL	Annular Ligament
TM	Tympanic Membrane (Eardrum)
TTT	Tensor Tympani Tendon
AML	Anterior Mallear Ligament
IMJ	Incudomallear Joint
ISJ	Incudostapedial Joint
